# Web-based psychoeducational interventions for managing cognitive impairment–a systematic review

**DOI:** 10.3389/fneur.2023.1249995

**Published:** 2023-09-13

**Authors:** Outi Vuori, Eeva-Liisa Kallio, Annamaria Wikström, Hanna Jokinen, Marja Hietanen

**Affiliations:** ^1^Department of Psychology and Logopedics, Faculty of Medicine, University of Helsinki, Helsinki, Finland; ^2^Division of Neuropsychology, HUS Neurocenter, Helsinki University and Helsinki University Hospital, Helsinki, Finland

**Keywords:** web-based, cognition, psychoeducation, rehabilitation, telerehabilitation, systematic review

## Abstract

**Objective:**

Web-based rehabilitation, a branch of telerehabilitation, is carried out over the internet, unrestricted by time or place. Even though web-based interventions have been reported as feasible and effective in cases of mood disorders, for example, such evidence on the effectiveness of web-based cognitive rehabilitation remains unclear. This systematic review summarizes current knowledge on web-based psychoeducational programs aiming to manage cognitive deficits in patients with diseases that affect cognition.

**Methods:**

Using the Ovid database and the Web of Science, we systematically searched the Cochrane Database of Systematic Reviews, Medline, and PsycINFO to identify eligible studies. The review protocol (CRD42021257315) was pre-registered with the PROSPERO International Prospective Register of Systematic Reviews. The search was performed 10/13/2022. Two reviewers independently screened titles, abstracts, and full-texts, and extracted data for the selected studies. Two independent reviewers assessed the methodological quality.

**Results:**

The search retrieved 6,487 articles. Four studies with different patient groups (stroke, traumatic brain injury, brain tumor, and cancer) met the inclusion criteria of this systematic review. The studies examined systematic cognition-focused psychoeducational rehabilitation programs in which the patient worked independently. Three studies found positive effects on subjective cognitive functions, executive functions, and self-reported memory. No effects were found on objective cognitive functions. However, the studies had methodological weaknesses (non-randomized designs, small sample sizes, vaguely described interventions). Overall, adherence and patient satisfaction were good/excellent.

**Conclusion:**

Web-based cognitive intervention programs are a new approach to rehabilitation and patient education. The evidence, although scarce, shows that web-based interventions are feasible and support subjective cognitive functioning. However, the literature to date is extremely limited and the quality of the studies is weak. More research with high-quality study designs is needed.

**Systematic review registration:**

https://www.crd.york.ac.uk/prospero/display_record.php?RecordID=257315, identifier: CRD42021257315.

## 1. Introduction

Digitalized health care services have the advantage of providing patients with access to treatment, irrespective of time and place (1–3). Web-based rehabilitation, a branch of telerehabilitation, is carried out at a patient's home over the internet. Online platforms and secure network connections also offer a new way to deliver cognitive and neuropsychological rehabilitation. The potential advantages of telerehabilitation in clinical practice are the possibility to offer services to larger population, reduce waiting times and to personalize rehabilitation but also to be cost-effectiveness (4). Still, the traditional way of carrying out neuropsychological rehabilitation is face-to-face at inpatient or outpatient clinics, but these services are regionally uneven and insufficient (5, 6).

Managing cognitive impairment in neurological disorders often requires intensive neuropsychological rehabilitation to improve cognitive functions as well as emotional and psychosocial wellbeing. A significant proportion of stroke patients show cognitive impairment despite good clinical recovery (7) and cognitive symptoms are also common after traumatic brain injury and encephalitis (8, 9). Rehabilitation for cognitive impairment has shown to be effective after brain injuries (10), and psychoeducation and compensatory strategy training (training of sets of conscious mental processes and techniques to compensate cognitive deficiencies) have been found to be the most efficient approaches for rehabilitation (11–13). Cognitive training (practice on a set of tasks designed to reflect particular cognitive functions) is also a common approach in cognitive rehabilitation, especially in online programs (14). Despite some near-transfer effect of attention and working memory training far-transfer and long-time effects of cognitive training are considered poor (15–17).

Psychoeducational framework is an established and essential approach originating from psychosocial treatment of psychiatry broadened to somatic diseases to provide support and information on the condition of patients and aims to improve functional abilities, mood, and quality of life (18, 19). Neurological patients benefit from sharing knowledge about symptoms, recovery, and symptom management (12, 20–22) and even patients with minor strokes have expressed the need for it after discharge (23). Information about stroke not only increases patients' understanding of the condition and its effects, but also enhances patients' contentment and diminishes depressive symptoms (21). Patients with mild cognitive symptoms also benefit from metacognitive and memory strategy training (11) and patients with mild traumatic brain injury cognitive strategy training was related to positive behavioral changes and better subjective and objective cognitive performance (24).

Considering the overlap and variety of the terminology in literature, in this review neuropsychological and cognitive rehabilitation is referred as broad neurocognitive rehabilitation. The interest in this study is in the neuropsychological or cognitive interventions combining psychoeducation (sharing knowledge) with cognitive strategy training (compensatory strategy training) leaving cognitive training interventions (practicing particular functions, “brain training”) outside when being the only approach of the intervention.

To date, the knowledge about structured web-based cognitive intervention programs, including psychoeducation and cognitive strategy training, is still scattered; only a few, mainly small-scale feasibility studies have been reported and deemed applicable to neurological patients (25–28). Web-based programs are also used to teach neurological patients self-management, but evidence of their effectiveness is limited (29). Web-based intervention programs have become evidence-based treatments for mood disorders (30), and have also been used for motor rehabilitation after stroke, for example (31). Yet, the effectiveness of cognitive or neuropsychological online rehabilitation programs is unclear.

The aim of this study was to systematically review the current knowledge on the effectiveness and feasibility of web-based psychoeducational interventions among adolescent and adult patients whose cognitive functions are affected by a somatic health condition.

## 2. Methods

This systematic review adhered to the Preferred Reporting Items for Systematic Reviews and Meta-analysis (PRISMA) (32). The review protocol (CRD42021257315) was pre-registered with the PROSPERO International Prospective Register of Systematic Reviews.

We used a PICO (population, intervention, comparison, outcome) framework to formulate the study design and search strategy. We asked: In adolescent or adult patients whose cognition is affected by a somatic health condition (P), are web-based psychoeducational interventions (I), in comparison to other interventions or no intervention at all (C), feasible and effective in improving subjective and/or objective cognitive functioning (O)? The search was targeted at adolescents or adults participating in a psychoeducational cognitive program or an intervention delivered remotely online and carried out independently by the patient. Subjective cognitive complaints, as evaluated by the patient's self-report (subjective cognitive functioning) and/or objective cognitive functions, as defined by performance in neuropsychological tests (objective cognitive functioning), were considered an outcome. We also considered data on adherence and program acceptability/feasibility.

### 2.1. Eligibility criteria

The trials were selected if they met the following criteria: (1) The intervention program was structured, delivered over the internet, and carried out by the patient independently; (2) The program focused on cognitive impairment; (3) The program included psychoeducation and cognitive strategic skill training; (4) The age group was from adolescence to working-aged participants; (5) The participants had a somatic health condition that affected their cognition; (6) The outcome was subjective and/or objective cognitive functioning.

Exclusion criteria were as follows: (1) The participants had a progressive neurodegenerative condition; (2) Solely cognitive training as approach; (3) The article was written in a language other than English; (4) Studies reported only the perspectives of health-care professionals or the future development of technology; (5) Studies reported only the feasibility of the programs.

Considering the novelty of the research field, no limitations were applied to sample sizes or study design, although we did primarily search for randomized controlled studies (RCTs). In addition to RCTs, we also included observational studies and single, one-arm studies without control groups. However, study protocol papers and case studies were excluded, as were abstracts and conference papers.

### 2.2. Information sources

The search was conducted in MEDLINE^®^, PsycINFO, the Cochrane Database of Systematic Reviews databases using the Ovid database search and the Web of Science database. Additional studies were identified from the reference lists of the relevant studies and accessed via the Google Scholar database. The initial search was performed in May 2021 and repeated in September 2021 and April 2022. The date of the last search was 10/13/2022. The searches were not subject to any time restrictions.

### 2.3. Search strategy

The search consisted of terms describing cognition or neurology, telehealth technology, and rehabilitation {e.g., [(web-based, internet-based or digital) and (cogniti^*^ or neuropsycholog^*^ or memory) and (rehabilitation or program^*^)]Ṫhe search strategy was adapted to the requirements of the databases searched. The full search strategy is included in Supplementary material. Search results were exported directly to EndNote X9 and duplicates were removed. We manually added additional identified records.

### 2.4. Selection process

**Figure 1:**
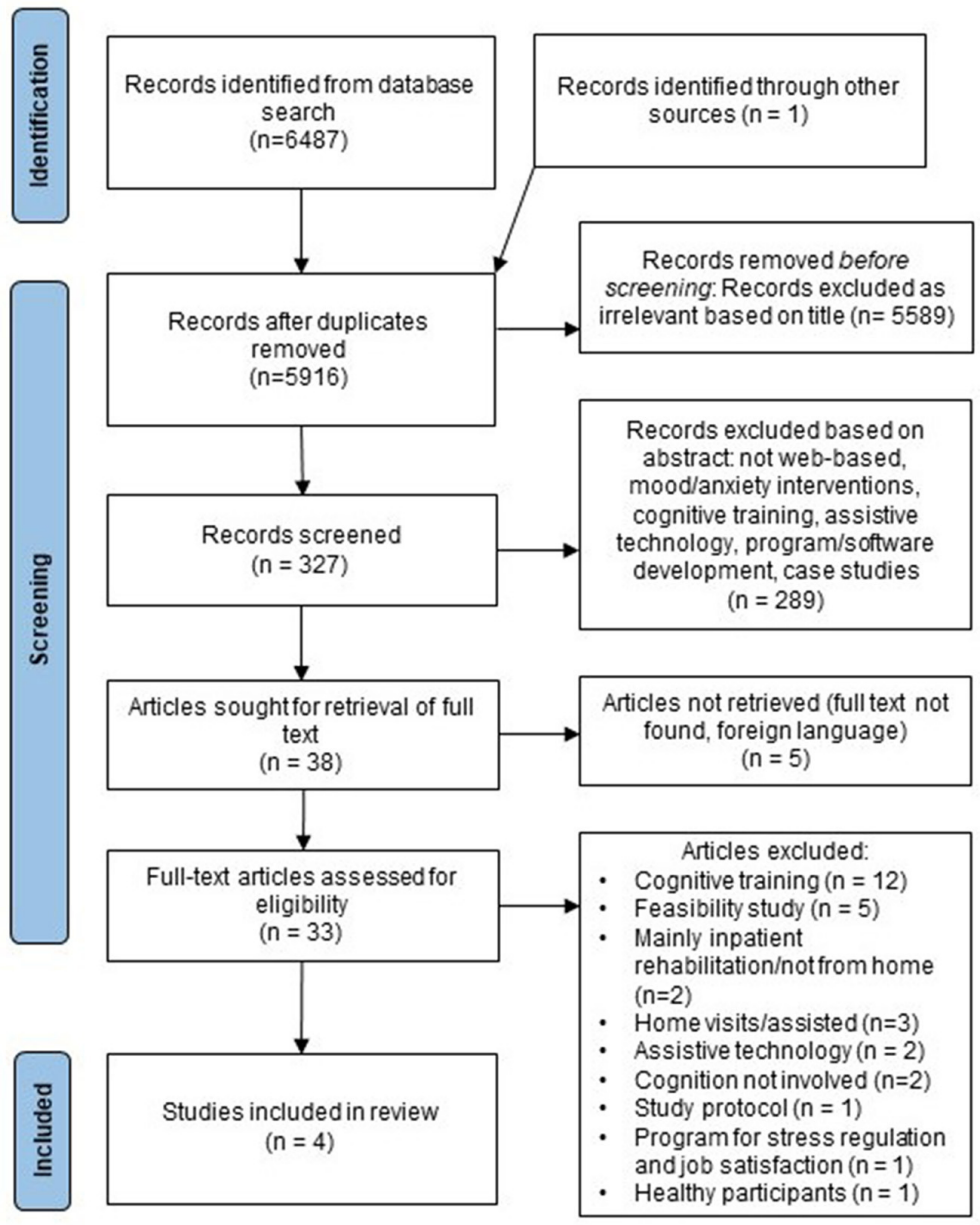
Flow chart of systematic review.

The screening process is described in the PRISMA flow diagram in Figure 1. Author OV conducted the screening. The titles of the identified papers were first reviewed for obvious exclusions. Abstracts were screened on the basis of their titles. If, after the abstract was read, it was unclear whether the article should be selected, the full text was reviewed. The selected full text articles were reviewed by authors OV and E-LK for eligible articles.

### 2.5. Quality assessment

Two reviewers (OV, E-LK) performed the quality assessment. Disagreements were discussed until consensus was reached. We applied the quality assessment tool created by Kallio et al. (33), which has previously been used to appraise research on cognitive training. In this rating system, the criteria is applied to randomized intervention trials used by Cochrane and collaborators (34) as well as the Delphi list (35), which is a criteria list for the quality assessment of randomized clinical trials.

The quality criteria are detailed in Table 1. Each criterion was worth 1 point. The methodological quality of the research was considered high when a study scored 8–10 points, while scores of 5–7 indicated moderate quality and scores <5 indicated low quality (24).

**Table 1:** Quality assessment.

**Study**	**1: Randomization method is performed**	**2: Inclusion and exclusion criteria are satisfactorily described**	**3. Groups are comparable at baseline**	**4: The study has sufficient statistical power to detect an effect (n > 25/group)**	**5: The intervention is adequately described**	**6: The measurements and outcome measures are valid and well defined**	**7: Those assessing the outcomes were blinded to the treatment allocation**	**8: Outcomes of the dropouts are described, and the analysis takes them into account**	**9: Intention-to-treat (ITT) analysis is applied**	**10: Appropriate statistical analyses are used**	**Total criteria met**
Babcock et al. (36) USA	-	+	-	-	+/-	+	-	-	n/a	+/-	2
Brouns et al. (37) The Netherlands	-	+	+	+	-	+	-	-	+	+	6
van der Linden et al. (38) The Netherlands	+	+	-	-	+/-	+	-	+	-	+/-	4
Mihuta et al. (39) Australia	+	+	+	+	+/-	+	-	+	+	+	8

+, Criterion fulfilled; +/-, criterion partly fulfilled; -, criterion not fulfilled.

## 3. Results

### 3.1. Studies

The initial search returned 6,487 records. Thirty-three full-text articles were assessed for eligibility and the screening process identified four eligible articles (Figure 1). The reviewers (OV, ELK) were in full agreement on which studies met the inclusion criteria. Two studies were RCTs with wait-list control groups (38, 39), one was a quasi-experimental study with an active control group (37) and one was a single-arm study without a control group (36). Due to a lack of studies and the variability of the interventions, we were unable to perform a meta-analysis on this data.

### 3.2. Participants

Table 2 presents the characteristics of the studies selected by the review. They included 452 participants in total, with the numbers of participants varying from 13 (36) to 318 (37). In three studies the participants were adults (37–39), and in one study adolescents (36). The mean age of the study participants ranged from 14 to 63. The participants in the intervention groups were heterogenous by diagnosis: stroke (37), TBI (36), brain tumor (38), and cancer patients (39).

**Table 2:** Study characteristics and main outcomes.

**Study**	**Intervention**	**Study design**	**Participants**	**Intervention methods**	**Assessments**	**Outcomes and effects**	**Adherence/ drop-out**
Babcock et al. (36) USA	Smart 4 weeks, 8 modules Independently	Pre-post design, single arm study *N* = 13 adolescents + parent	Age 14.3 TBI (max 96 h from occurrence)	Symptom monitoring, activity tracking, skill training, feedback, psychoeducation	Baseline, follow-ups 1, 2, 4 weeks	Outcome (measure): *primary* symptom burden (HBI), functional disability (FDI), executive functioning (brief-BRIEF), *secondary* behavior problems (CBCL, YSR), concussion knowledge (CDC), symptom monitoring (PCSS) Effect: *primary* significant improvement in functional disability and executive functions [parent-rated FBI, 0.5 (1.2), *n* = 11, *p* = 0.009, parent-rated brief-BRIEF 31.8 (7.2), *n* = 11, *p* = 0.03], significant improvement in symptom burden [parent-rated HBI 7.9 (7.5), *n* = 11, *p* = 0.004, self-report HBI 9.7 (10.1), *n* = 9, *p* = 0.0005] *secondary* no significant changes	100/38%
Brouns et al. (37) The Netherlands	Fast@home 16 weeks, 4 modules Some independently, some assisted (not specified)	Quasi-experimental study *N* = 318 [IG *N* = 165 (eRehab addition to conventional rehabilitation), CG = 54] [conventional rehabilitation]	Age 62.6 (IG), 58.6 (CG) Stroke (max 6 m from occurrence)	Cognitive and physical exercises, activity tracking, psychoeducation	Baseline, follow-ups 3 + 6 m	Outcome (measure): *primary* disability (SIS) *secondary* quality of life (EQ5D), mental and physical health (SF-12), fatigue (FSS), self-management (PAM-13), participation (USER-P), physical activity (IPAQ-SF) Effect: *primary* significant improvement in communication [SIS IG: 88.9 (12.1), *n* = 54, CG: 87.4 (8.9), *n* = 153, *p* = 0.019], memory [SIS IG: 87.7 (11.5), *n* = 54 CG: 82.1 (10.3), *n* = 153, *p* = 0.031], meaningful activities [(only change scores reported) SIS IG: 16.2 (17.2), *n* = 54, CG: 1.6 (19.2), *n* = 153, *p* = 0.040], and physical strength [SIS IG: 69.2 (10.4), *n* = 54, CG:67.6 (22.4) *n* = 153, *p* = 0.008] at 6 m follow-up *secondary* no significant changes	n/a
van der Linden et al. (38) The Netherlands	ReMind 10 weeks, 6 modules Independently, telephone checkup every 2 weeks	RCT *N* = 45 [IG *N* = 20 CG *N* = 25 (wait list, no other rehabilitation)]	Age 45.7 (IG), 52.6 (CG) Brain tumor [low grade glioma, meningioma] (3 m after surgery)	Psychoeducation, strategy training, attention retraining	Baseline, follow-ups 3 + 9 m	Outcome (measure): *primary* objective cognitive performance (CNS VS, WAIS-III digit span, verbal fluency) *secondary* subjective cognitive functioning (CFQ), executive functions (BRIEF-A), fatigue (MFI-20), mood (HADS) *tertiary* enrolment, attrition, adherence, patient satisfaction Effect: *primary* no significant changes *secondary* no significant changes *tertiary* recruitment difficulties, patient satisfaction good/excellent	85–91/21%
Mihuta et al. (39) Australia	EReCog 4 weeks, 4 modules Independently	RCT *N* = 76 [IG *N* = 40 CG *N* = 36 (wait-list, no other rehabilitation)]	Age 55.1 (IG), 56.9 (CG) Cancer (96.9% breast cancer, primary treatment completed min. 6 m ago)	Strategy training, relaxation, exercises, discussion, psychoeducation	Baseline, follow-ups 4 w + 3 m	Outcome (measure): *primary* subjective cognitive functioning (FACT-cog-3) *secondary* additional subjective cognitive functioning (BAPM), objective cognitive functioning (WebNeuro), psychosocial wellbeing (KPDS, BIPQ, EORTC-QLQ-C30), program satisfaction Effect: *primary* non-significant trend in improving perceived cognitive impairment [IG: 50.5 (12.7), *n* = 32, GC: 48.2 (15.0), *n* = 33, *p* = 0.089] *secondary* significant reduction in self-reported prospective memory failures at post-treatment [IG: 1.78 (0.46), *n* = 32, GC: 1.79 (0.53), *n* = 33, *p* < 0.05] and 3 m follow-up [IG: 1.67 (0.52), *n* = 32, GC: 1.66 (0.50), *n* = 33, *p* = 0.025], program satisfaction good/excellent	87/13%

HBI, Health and Behavior Inventory (range 20–80, low score = better health/behavior); FDI, Functional Disability Inventory (range 0–60, low score = better functioning); Brief-BRIEF, 24-item Behavior Rating Inventory of Executive Functioning (range 24–72, low score = better behavior); CBCL, Child Behavioral Checklist (range 0–226, low score = better behavior); YSR, Youth Self Report (range 0–224, low score = better behavior); CDC Head's Up Concussion quiz (range 0–11, high score = better knowledge); PCSS, Post-concussion symptom scale (range 0–126, low score = better health); SIS, Stroke Impact Scale (range 0–100, high score = better performance); EQ5D, EuroQol-5D-3L (range 1–15, low score = better health); SF-12, Short-Form Health Survey (range 0–100, low score = better health); FSS, Fatigue Severity Scale (range 9–63, low score = better functioning); PAM-13, Patient Activation Measure Short Form 13 (0–100, low score = better self-management); USER-P, Utrecht Scale for Evaluation of Rehabilitation-Participation (range 0–100, low score = better participation); IPAQ-SF, International Physical Activity Questionnaire Short Form (range 0 <, high score = better activity); CNS VS, Central Nervous System Vital Signs (standardized z-scores, range >-2, 5-2, 5 <, high score = better performance); WAIS-III, Wechsler Adult Intelligence Scale 3^*rd*^ version (standardized z-scores, range >-2, 5-2, 5 <, high score = better performance); CFQ, The Cognitive Failure Questionnaire (range 0–100, low score = better functioning); BRIEF-A, Behavior Rating Inventory of Executive Function (range 0–100, low score = better behavior); MFI-20, Multidimensional Fatigue Inventory (range 20–100, low score = better functioning); HADS, Hospital Anxiety and Depression Scale (range 0–21, low score = better health); FACT-Cog-3, Functional Assessment of Cancer Therapy—Cognitive Scale (range 0–148, high score = better performance); BAPM, Brief Assessment of Prospective Memory (range 0–5, low score = better functioning); WebNeuro (standardized z-scores, range >-2, 5-2, 5 <, high score = better performance); KPDS, Kessler Psychological Distress Scale (range 10–50, low score = better health); BIPQ, Brief Illness Perception Questionnaire (range 0–80, low score = better functioning); EORTC-QLQ-C30, European Organization for Research and Treatment of Cancer Quality of Life Questionnaire (range 0–100, high score = better quality of life).

### 3.3. Interventions

In two studies (RCTs) the intervention protocol had been described in previous papers (28, 40). The interventions lasted 4–16 weeks, but the data on the frequencies of the sessions or the total duration of the interventions were lacking or unclearly described in all the studies.

As required by the inclusion criteria, psychoeducation was included in all the studied interventions, and it was combined with strategy training (38, 39), strategy exercises outside the program (36, 39), and cognitive training (37). In one study (27) the strategy training was fill-in exercises within the program. Execution or form of the strategy training or exercises in other studies was not reported (28, 30). Studies with exercises outside the program did not report if completing the exercises outside the program was monitored in some way (28, 30). A physical activity tracker, exercises (36, 37) and relaxation were also used (39).

All the studies described the contents of the interventions on a general level, and contents was divided into different themed modules. The availability of the modules varied. In one study, availability was dependent on symptom burden (36). In the study by Mihuta et al. (39), completing each module before continuing to the next was compulsory. In the study by Brouns et al. (37), the psychoeducation module was reportedly available to all the participants and the other modules (cognitive training, physical exercises) were tailored individually, although how this was done was not reported. One study did not report on the availability of the modules or contents (38). The intervention for adolescents was also open to their parents (36).

Two interventions were conducted independently, with reminder emails (36, 39). In one study, the researcher made telephone checkups every 2 weeks (38). One intervention was conducted alongside conventional rehabilitation and did not report on the therapist's role in the web-based intervention (37). Two interventions were used as an application on a tablet (37, 38), and the others via an internet website (36, 39).

### 3.4. Outcomes/effects

Table 2 presents all the outcome measures and effects. The main outcome measures were subjective cognitive functioning (36–39), objective cognitive functioning (38, 39) and psychological wellbeing (39). Symptom monitoring (36), fatigue (37, 38), and satisfaction with the program (38, 39) were also evaluated. One study of adolescent TBI patients also included parent-rated evaluation (36).

Some self-reported improvements were found in subjective cognitive functioning. The study of adolescent TBI patients reported a significant improvement after the intervention in self-reported measure of functional/physical abilities and parent-rated measure to assess executive functions (36). A study of stroke patients found significant improvement in self-reported stroke impact scale assessing communication, memory, and meaningful activities at 6-month follow-up (37). A significant reduction in self-reported measure of prospective memory failures at post-treatment and 3-month follow-up was found in a study of cancer patients (39). Same study reported also a non-significant trend in decreasing subjective perceived cognitive impairment (39).

No effects on objective cognitive performance were found post treatment (38, 39). However, one study found a significant difference in favor of the intervention in a computerized neuropsychological test battery 1 year after brain tumor surgery (38).

One study reported a significant decrease in adolescents' self-reported and parent-rated TBI symptom burden (36). No significant differences were found in fatigue (37, 38).

### 3.5. Adherence to and satisfaction with program

Completion rate of the web-based interventions was high in three studies; 85–100% of the participants who started the intervention program also completed it (36, 38, 39). The adherence to exercises was high (85–91%) in one study (27). One study did not report the adherence rate (37). Satisfaction with the program was described as good or excellent in two studies (27, 28) and participants' appreciation and satisfaction with web-based intervention was 7.7 on a 10-point scale in one study (39).

### 3.6. Quality assessment

As shown in Table 1, only one of the selected studies was rated as a high-quality study (39). One study were considered to be of moderate quality (37) and two studies to be low quality (36, 38). Two of the four studies were not RCTs and did not fulfill the intervention description criterion (36, 37). Notable methodological limitations were small sample sizes (*n* < 25/group) and the incomparability of the groups at baseline (36, 38). All the studies failed to meet the criteria with blinding.

## 4. Discussion

The aim of this systematic review was to collect and evaluate the current evidence on the effectiveness and feasibility of web-based psychoeducational interventions combined with cognitive strategy training for managing cognitive impairment.

Overall, to date, the literature on digitalized cognitive or neuropsychological rehabilitation is very limited and only four studies fulfilled the inclusion criteria. Two of these studies were RCTs (38, 39), one a quasi-experimental study (37) and one a single-arm study (36). The psychoeducational content of the interventions was commonly combined with cognitive strategy training (36, 38, 39). The studies were heterogeneous in terms of age, diagnosis, and design. The diversity and heterogeneity of the interventions and populations in selected studies may hinder the comparison.

Sporadic findings in this review suggest that web-based cognitive interventions may improve self-reported subjective cognitive functioning. At 6-month follow-up, the study of stroke patients showed self-evaluated improvement in communication, memory, and meaningful activities in favor of the intervention group (37). Patients with cognitive impairment after oncological treatment showed a significant reduction in self-reported prospective memory failures post treatment and 3-month follow-up (39). The TBI adolescents self-reported recovery of functional/physical disability and executive functions parent-rated after the intervention (36). However, the study did not have a control group. Two studies indicated that some of the rehabilitation effects were maintained for longer thanks to the web-based intervention (37, 38).

On the basis of this review, the web-based interventions had no effects on objective cognitive functioning (neuropsychological test performance). However, in one study, at 9-month follow-up, fewer brain tumor participants showed cognitive impairment in the intervention group (38). The authors emphasized the uncertainty of the finding but cautiously propose that the intervention program had small beneficial effects (38).

Profound methodological problems were found in the quality of the designs of the selected studies (see Table 1). Only two studies were RCTs and only one of these was assessed as high quality. Most studies had small sample sizes and in all the studies the size of the intervention groups was under 54. In two studies, sampling was done through self-selection, which might result in biased selection—-as participants might be more motivated to take part in rehabilitation activities. Studies used self-reported outcome measures which might prone to bias to willingness to please. These might have led to an increased risk of positive findings. In addition, in some cases, information and precise descriptions of the interventions were lacking according to the Template for Intervention Description and Replication (TIDieR) checklist (41). The TIDieR checklist is recommended for use in intervention studies to describe the intervention for good reporting policy (42).

In addition to methodological issues, studies selected in this systematic review sets few notable limitations to larger scale conclusions. Due to data reported in the studies, moderation analysis and recommendations of populations benefitting from web-based interventions could not be made. There are also lack of comparison with other interventions which leaves unclear whether web-based rehabilitation programs are superior to other intervention approaches. Also, sustainability of the effects remains unclear only one study having over 6-month follow-up (27). In all, the ability to generalize from these studies remain dubious.

A few interesting studies arose that did not fulfill the inclusion criteria. A web-based program for cognitive aging of healthy adults (excluded for not having somatic condition affecting cognition) had small to moderate effects on the self-reported feeling of stability in memory functioning and locus of control over memory in an RCT study design (43). Participants also reported fewer cognitive mistakes, less worry about cognition and dementia, and better ability to cope with cognitive loads. EpilepsyJourney, a web-based program for adolescent epilepsy patients with cognitive symptoms and behavioral problems, was believed to improve executive functions and emotional and behavioral functioning in a pre-post design study (44). The program consisted of problem-solving interventions with psychoeducational modules and support from a health care professional via video (excluded for not carrying out independently). The results of these studies could be interpreted as parallel to the sporadic findings reported in this review.

In all, despite methodological flaws, the psychoeducational components of the interventions may have contributed to the increased feeling of control over subjective cognitive functioning and may have alleviated symptom-induced anxiety—the participants received reliable information about their cognition and how to manage cognitive deficits, which is believed to be effective and necessary among neurological patients (11–13, 23).

Although the evidence of the effectiveness of web-based interventions to date is scarce, in this review they were found to be a feasible approach to arranging cognitive rehabilitation, as in previous studies (26–28, 30). It seems that completion and adherence to web-based rehabilitation may be good or even excellent in patient groups with cognitive deficits. Web-based interventions were also considered to be safe, as no adverse outcomes of TBI symptoms were reported (36).

While this review followed robust methodology and a systematic search strategy to identify relevant trials, it does have limitations. We restricted our search to English language publications only, which may have excluded some relevant studies. Despite the voluminous search strategy, the search terms used might have led to the exclusion of some interesting studies due to the novelty of the field and the as-of-yet unestablished terminology related to telerehabilitation solutions. We also relied on published reports only, which may lead to publication bias.

Web-based interventions for neurological patients have several benefits. They have the potential to reach large populations and to be used widely—-accessibility and adherence is excellent and irrespective of time, and they can even be conducted at home (only a technical device with an internet connection is needed). As the aging of the population and shortage of health care resources increases, interventions carried out independently online will become more essential. Overall, the use of telerehabilitation services will increase in future healthcare, and thus we need evidence of their advantages and weaknesses. If proven to be effective, telerehabilitation services may also broaden the variety of neuropsychological interventions and have the potential to equalize regional differences, make rehabilitation more cost-effective (4), and reduce waiting times for rehabilitation services.

### 4.1. Conclusion

To our knowledge, this is the first systematic review on psychoeducational web-based intervention programs for cognitive deficits in patient populations with cognitive impairment due to an injury or a treatment that affects brain functions. According to the evidence of this review, it appears that adolescent and working-aged patients are able and willing to use web-based psychoeducational programs, and that these interventions may increase patients' sense of control over their cognitive functioning. However, research on intervention studies in telerehabilitation is only in its early stages and therefore evidence of its effectiveness is still very limited and weak. Well-designed web-based intervention studies are crucial for increasing the evidence-base of this new research area, which is extremely contemporary and cautiously promising.

## Data availability statement

The original contributions presented in the study are included in the article/Supplementary material, further inquiries can be directed to the corresponding author.

## Author contributions

OV: drafting the manuscript. OV, E-LK, AW, and HJ: writing the final version. E-LK, AW, HJ, and MH: supervision. OV and E-LK: selection of studies and quality assessment of studies. All authors: conception of the manuscript. All authors have read and approved the final version of the manuscript.
