# Supplementary material for: Web-based psychoeducational interventions for managing cognitive impairment–a systematic review
Source: Front Neurol. 2023 Sep 13;14:1249995. doi: 10.3389/fneur.2023.1249995 (PMC10535106; doi:10.3389/fneur.2023.1249995)
Supplement: Supplementary file 1 [file Table_1.DOCX]

Supplementary Material

Web-based psychoeducational interventions for managing cognitive impairment – a systematic review

Outi Vuori*, Eeva-Liisa Kallio, Annamaria Wikström, Hanna Jokinen, Marja Hietanen

*** Correspondence:** Outi Vuori: outi.vuori@helsinki.fi

# Search strategy

| **Cochrane Database of Systematic Reviews** | | | | |
| --- | --- | --- | --- | --- |
|  | neuropsycholog* | | AND | home-based |
|  | cogniti* |  |  | web-based |
|  | memory |  |  | internet-based |
|  |  |  |  |  |
|  | neuropsycholog* | | AND | home-based |
|  | cogniti* |  |  | web-based |
|  | memory |  |  | internet-based |
|  |  |  |  | telerehabilitation |
|  |  |  |  | eHealth |
|  |  |  |  |  |
| **Medline** |  | Combinations of keywords and free text terms | | |
| MesH Keywords | |  |  |  |
|  | internet/ or internet-based intervention/ | | | |
|  | Mobile Applications/ | | |  |
|  | Cognition Disorders/ | | |  |
|  | brain diseases/ or brain injuries/ | | | |
|  | Stroke/ |  |  |  |
|  | neurological rehabilitation/ or stroke rehabilitation/ or telerehabilitation/ | | | |
|  | Therapy, Computer-Assisted/ | | |  |
| Free text terms | |  |  |  |
|  | ((web-based or internet-based) and (cogniti* or neuropsycholog* or memory) and rehabilitation) | | | |
|  | ((web-based or internet-based or digital) and (cogniti* or neuropsycholog* or memory) and rehabilitation) | | | |
|  | ((web-based or internet-based or digital) and (cogniti* or neuropsycholog* or memory) and (rehabilitation or program*)) | | | |
|  | ((cogniti* or neuropsycholog* or memory) and telerehabilitation) | | | |
|  | (digital and (cogniti* or neuropsycholog* or memory) and (rehabilitation or program*)) | | | |
|  |  |  |  |  |
|  | web-based cognitive rehabilitation.ab,kf,ti. | | | |
|  | "Web-based cognitive rehabilitation program*".ab,kf,ti. | | | |
|  | web-based neuropsychological rehabilitation.ab,kf,ti. | | | |
|  | cognitive telerehabilitation.ab,kf,ti. | | | |
|  | cognitive telerehabilitation program.ab,kf,ti. | | | |
|  | neuropsychological telerehabilitation.ab,kf,ti. | | | |
|  | neuropsychological telerehabilitation program.ab,kf,ti. | | | |
|  | internet-based cognitive rehabilitation.ab,kf,ti. | | | |
|  | internet-based neuropsychological rehabilitation.ab,kf,ti. | | | |
|  | internet-based cognitive rehabilitation program.ab,kf,ti. | | | |
|  | internet-based neuropsychological rehabilitation program.ab,kf,ti. | | | |
|  | neuropsychological ehealth.ab,kf,ti. | | | |
|  | cognitive ehealth.ab,kf,ti. | | |  |
|  | teleneuropsychology.ab,kf,ti. | | |  |
|  | teleneuropsychology rehabilitation.ab,kf,ti. | | | |
|  | digital neuropsychological rehabilitation.ab,kf,ti. | | | |
|  | digital cognitive rehabilitation.ab,kf,ti. | | | |
|  | web-based memory rehabilitation.ab,kf,ti. | | | |
|  | internet-based memory rehabilitation.ab,kf,ti. | | | |
|  | digital memory rehabilitation.ab,kf,ti | | | |
|  |  |  |  |  |
| **PsycINFO** |  | Combinations of keywords and free text terms | | |
| Keywords | |  |  |  |
|  | internet/ |  |  |  |
|  | mobile applications/ | | |  |
|  | cognitive impairment/ or brain damage/ or brain injuries/ or neurocognitive disorders/ | | | |
|  | brain disorders/ | |  |  |
|  | cerebrovascular accidents/ | | |  |
|  | neuropsychological rehabilitation/ or neurorehabilitation/ | | | |
|  | cognitive rehabilitation/ | | |  |
|  | telerehabilitation/ | |  |  |
|  | computer assisted therapy/ | | |  |
| Free text terms | |  |  |  |
|  | Web-based cognitive rehabilitation.ab,mh,ti. | | | |
|  | "Web-based cognitive rehabilitation program*".ab,mh,ti. | | | |
|  | web-based neuropsychological rehabilitation.ab,mh,ti. | | | |
|  | cognitive telerehabilitation.ab,mh,ti. | | | |
|  | cognitive telerehabilitation program.ab,mh,ti. | | | |
|  | neuropsychological telerehabilitation.ab,mh,ti. | | | |
|  | neuropsychological telerehabilitation program.ab,mh,ti. | | | |
|  | internet-based neuropsychological rehabilitation.ab,mh,ti. | | | |
|  | internet-based cognitive rehabilitation.ab,mh,ti. | | | |
|  | internet-based cognitive rehabilitation program.ab,mh,ti. | | | |
|  | internet-based neuropsychological rehabilitation program.ab,mh,ti. | | | |
|  | neuropsychological ehealth.ab,mh,ti. | | | |
|  | cognitive ehealth.ab,mh,ti. | | |  |
|  | teleneuropsychology.ab,mh,ti. | | |  |
|  | teleneuropsychology rehabilitation.ab,mh,ti. | | | |
|  | digital neuropsychological rehabilitation.ab,mh,ti. | | | |
|  | digital cognitive rehabilitation.ab,mh,ti. | | | |
|  | web-based memory rehabilitation.ab,mh,ti. | | | |
|  | internet-based memory rehabilitation.ab,mh,ti. | | | |
|  | digital memory rehabilitation.ab,mh,ti. | | | |
|  | ((web-based or internet-based) and (cogniti* or neuropsycholog* or memory) and rehabilitation).ab,ti,mh. | | | |
|  | ((web-based or internet-based or digital) and (cogniti* or neuropsycholog* or memory) and rehabilitation).ti,ab,mh. | | | |
|  | ((web-based or internet-based or digital) and (cogniti* or neuropsycholog* or memory) and (rehabilitation or program*)).mh,ti,ab. | | | |
|  | ((cogniti* or neuropsycholog* or memory) and telerehabilitation).ab,ti,mh. | | | |
|  | (digital and (cogniti* or neuropsycholog* or memory) and (rehabilitation or program*)).mh,ti,ab. | | | |
|  |  |  |  |  |
| **Web of Science** | |  | Combinations of free text terms | |
|  | memory |  |  |  |
|  | digi* |  |  |  |
|  | rehabilit* |  |  |  |
|  | web-based | |  |  |
|  | internet-based | |  |  |
|  | home-based | |  |  |
|  | ehealth |  |  |  |
|  | telerehabilit* | |  |  |
|  | teleneuropsychol* | |  |  |
|  | cogniti* |  |  |  |
|  | neuropsychol* | |  |  |
